# Trace Amine-Associated Receptor 1 Agonist Modulates Mismatch Negativity-Like Responses in Mice

**DOI:** 10.3389/fphar.2019.00470

**Published:** 2019-05-03

**Authors:** Aleksander A. Aleksandrov, Veronika M. Knyazeva, Anna B. Volnova, Elena S. Dmitrieva, Nadezhda V. Polyakova, Raul R. Gainetdinov

**Affiliations:** ^1^Department of Higher Nervous Activity and Psychophysiology, Saint Petersburg State University, Saint Petersburg, Russia; ^2^Department of General Physiology, Saint Petersburg State University, Saint Petersburg, Russia; ^3^Institute of Translational Biomedicine and Saint Petersburg University Hospital, Saint Petersburg State University, Saint Petersburg, Russia

**Keywords:** trace amine-associated receptors, TAAR1, RO5263397, mismatch negativity, event-related potentials, oddball paradigm, schizophrenia biomarkers

## Abstract

The trace amine-associated receptor 1 (TAAR1) is a G protein-coupled receptor widely expressed in the mammalian brain, particularly in limbic system and monoaminergic areas. It has proven to be an important modulator of dopaminergic, serotoninergic, and glutamatergic neurotransmission and is considered to be a potential useful target for the pharmacotherapy of neuropsychiatric disorders, including schizophrenia. One of the promising schizophrenia endophenotypes is a deficit in neurocognitive abilities manifested as mismatch negativity (MMN) deficit. This study examines the effect of TAAR1 partial agonist RO5263397 on the MMN-like response in freely moving C57BL/6 mice. Event-related potentials (ERPs) were recorded from awake mice in the oddball paradigm before and after RO5263397 administration. The RO5263397 (but not saline) administration increased the N40 amplitude in response to deviant stimuli. That provided the MMN-like difference at the 36–44 ms interval after the injection. The pitch deviance-elicited changes before the injection and in the control paradigm were established for the P68 component. After TAAR1 agonist administration the P68 amplitude in response both to standard and deviant stimuli was increased. These results suggest that the MMN-like response in mice may be modulated through TAAR1-dependent processes (possibly acting through the direct or indirect glutamate NMDA receptor modulation), indicating the TAAR1 agonists potential antipsychotic and pro-cognitive activity.

## Introduction

Trace amines (tyramine, tryptamine, phenylethylamine, and octopamine) are related by structure to classical biogenic amines and have long been known to be present in low concentrations (0.1–10 nM) in the mammalian nervous system. Trace amines (TAs) dysregulation is associated with psychiatric impairments, including the affective behavior, schizophrenia, depression, Parkinson’s disease, and attention deficit hyperactivity disorder (Branchek and Blackburn, 2003; Burchett and Hicks, 2006; Berry et al., 2017; Gainetdinov et al., 2018). For example, the increased levels of phenylethylamine, tyramine, and tryptamine levels in urinary and plasma of schizophrenic patients suggest the TA role in the pathophysiology of the disease (Davis, 1989; Berry, 2007).

Although TAs have been investigated for a long time (Boulton, 1974), their receptors in vertebrates are relatively recently discovered (Borowsky et al., 2001). The so-called trace amine associated receptors (TAARs) belong to the family of G protein-coupled receptors and are divided into three subgroups: TAAR 1–4, TAAR 5 and TAAR 6–9 (Lindemann et al., 2005).

Trace amine associated receptor1 is the best studied member of the TA receptor family (Lindemann et al., 2008; Bradaia et al., 2009; Revel et al., 2011; Leo et al., 2014). It is found in the central nervous system of rodents, monkeys and humans and is activated by trace amines, such as phenylethylamine and tyramine (Miller, 2011). TAAR1 is mainly expressed in the limbic system and monoaminergic systems regions, including the hypothalamus, substantia nigra, dorsal raphe nucleus (DRN), and ventral tegmental area (VTA), amygdala, nucleus of the solitary tract, rhinal cortices and subiculum (Lindemann et al., 2008; Panas et al., 2010).

Several studies have established that TAAR1 is localized in dopaminergic and serotonergic regions of the brain and acts as a neuromodulator for dopaminergic, serotonergic and glutamatergic systems. Experiments *in vitro* with TAAR1 agonists and antagonists have revealed that TAAR1 reduces the dopamine (DA) and serotonin [5-hydroxytryptamine (5-HT)] neurons firing frequency in limbic and monoaminergic systems, such as VTA and DRN (Bradaia et al., 2009; Revel et al., 2011; Espinoza et al., 2011). TAAR1 agonists administration *in vivo* have been also known to reduce the firing rate of DA neurons and inhibit the behavioral and electrophysiological effects of psychostimulants (Revel et al., 2011; Revel et al., 2013). Indeed, *Taar1* knockout mice have no overt phenotype, however, exhibiting schizophrenia-like properties. This is manifested in an elevated amphetamine-induced hyperactivity and an increased striatal release of DA, norepinephrine and 5-HT (Wolinsky et al., 2007; Lindemann et al., 2008); a D2 DA receptor supersensitivity in the striatum (Hirvonen et al., 2005; Corripio et al., 2006, 2011); a decreased function of N-methyl-D-aspartate (NMDA) receptors in the prefrontal cortex (Espinoza et al., 2015) and a deficit in the prepulse inhibition test (Wolinsky et al., 2007; Polyakova et al., 2018). Taken together, these data suggest that TAAR1 agonist is an important negative monoaminergic and positive glutamatergic neurotransmission modulator, which makes it a potential useful target for the pharmacotherapy of neuropsychiatric disorders, including schizophrenia.

Common schizophrenia symptoms include a deficit in several neurocognitive abilities, which is manifested in an impaired prepulse inhibition, a P50 event-related potential (ERP) suppression, a deficit in mismatch negativity (MMN) and P300. Disturbances in these processes reflect the deficient ability to regulate the environmental information inflow and are highly replicated schizophrenia biomarkers (Turetsky et al., 2007; Light and Swerdlow, 2015).

Mismatch negativity is a differential auditory ERP response to stimuli that deviate from repeatedly presented standard stimuli and reflect automatic and pre-attentive auditory stimulus-discrimination processes (Näätänen et al., 1978, 2011). A gradual decrease in MMN amplitude reflects cognitive and functional impairments that depend on the schizophrenia disease endurance (Javitt et al., 1993; Michie, 2001; Light and Braff, 2005; Näätänen and Kähkönen, 2009). Neuropharmacological basis of these violations is not fully understood. Among the major neurotransmitter systems glutamate-, 5-HT- and dopaminergic systems are known to be involved in the MMN generation. Therefore, the study of TAAR1 contribution to the pharmacological regulation of these processes may help to understand the neurophysiological basis of schizophrenia and develop new treatments to this debilitating disorder.

Here, we investigated the selective TAAR1 partial agonist RO5263397 effects on the MMN-like response in the oddball paradigm in freely moving C57BL/6 mice.

## Materials and Methods

### Animal Preparation

Twenty-two male C57BL/6 mice (weight range 25–40 g) were obtained from the vivarium of the Institute of Translational Biomedicine (Saint Petersburg State University, Saint Petersburg, Russia).

All animal studies were carried out in strict accordance with the guidelines of the Ministry of Health of Russian Federation and the principles of the Basel Declaration. All experiments were approved by the Saint Petersburg State University Ethical Committee for Animal Research (approval number 131-03-4 from March 14, 2016). All surgical procedures were performed under anesthesia, and all efforts were made to minimize suffering.

Mice were anesthetized with Zoletil (150 mg/kg i.p.) and xylazine (0.2 mg/kg i.m.). Epidural active electrodes were implanted above the two auditory cortices (bregma AP = -3.5; L = ± 3.5). Two reference electrodes were located at AP = + 2.0, L = ± 0.5; the ground electrode was located at AP = -1.0, L = -0.5. The leads were fixed with dental cold-cured plastic Acrodent (JSC Ctoma, Ukraine). The operation field was treated with Baneocin. Baytril^®^ (enrofloxacin, 2.5 mg/kg i.m.) that was administered preoperatively as an antibiotic. The animals were allowed to recover for at least 4 days after surgery. The electrode placement was confirmed after the experiment.

### Experimental Design

Electrocorticogram (ECoG) was recorded continuously during the experiment by a Mitsar-EEG-05/70-201 amplifier with WinEEG software (v. 2.4, Mitsar Co., Ltd., Russia) within the experimental chamber located between the speakers. Each animal experiment was conducted with a 1-day break. The awake mice were placed in the experimental chamber for 15 min before the ERPs session and were free to explore during recordings. After that, the first ERP recording in the oddball paradigm was performed; RO5263397 (1 mg/kg i.p.) or 0.9% NaCl were administered 15 min prior to the start of the second ERP recording session. RO5263397 was prepared in 0.9% saline solution before the injection. The drug dose was selected based on the previous studies of the RO5263397 dose-dependent effect (Revel et al., 2013). The order of conditions was counter balanced across the animals.

### Data Analysis

Electrocorticogram was filtered (bandpass 0.32–70 Hz), and artifacts were removed by visual inspection during offline analysis. High frequency (100–200 Hz) and high amplitude waves (amplitude exceeds ± 500 μV on any of the channels) occurred primarily during the grooming (Roger et al., 2009) and were rejected.

Stimuli were presented in oddball and reverse oddball paradigms via speakers by the Psytask software (v. 2.4, Mitsar Co., Ltd., Russia). Standard and deviant sinusoidal tones were 100 ms in length (including 5 ms of rise/fall time), and the intensity was 86 dB SPL averaged across the experimental chamber. Standards were 6 or 8 kHz frequency tones (probability 85%), and deviants were 8 or 6 kHz frequency tones (probability 15%) presented in a pseudo-random order with 500 ISI such that before each deviant stimulus, at least one standard stimulus occurred. The order of paradigms was counter balanced across animals. Data analysis was performed according to Featherstone et al. (2015). ERP to standard and deviant tones were quantified. Baseline data were collected from – 100 to 0 ms before stimulus onset. To receive resulting ERPs from the equal number of standard and deviant tones, only the last standard tone prior to the deviant was averaged. Finally, a difference wave was calculated as the deviant minus standard ERP. Mean latency and amplitude measures (baseline to peak) were extracted over 36–44 ms and 68–100 ms latency windows corresponding to the N40 and P68 ERP peaks. Mean latencies and amplitudes were obtained for standard, deviant, and difference responses.

All data were normally distributed according to the Kolmogorov–Smirnov test. Mean latencies and amplitudes of the ERP components were analyzed using three-way repeated measures analysis of variance (rmANOVA) with Fisher LSD *post hoc* test (IBM SPSS Statistic v. 21, IBM Corporation, New York, United States) with the factors *Injection* (before and after), *Stimulus type* (deviant and standard), and *Electrode location* (left and right). The Greenhouse–Geisser correction was used where sphericity assumptions were violated. In addition, paired sample *t*-test was applied. Difference MMN-like responses were analyzed using two-way rmANOVA with the factors *Injection* (before and after) and *Electrode location* (left and right).

## Results

**FIGURE 1:**
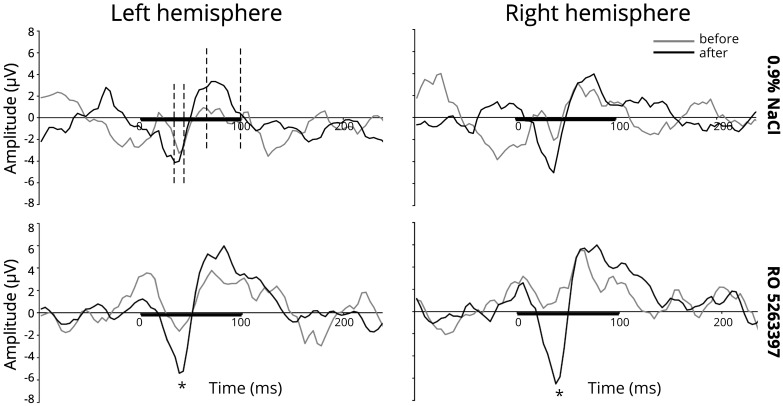
MMN-like response (differential component) above the left and right auditory cortex before (gray line) and after (black line) 0.9% NaCl or 1 mg/kg RO 5263397. The black line on the *x*-axis shows a stimulus duration. The dashed lines are the intervals taken for the amplitudes and latencies analysis. Stars denote statistical significance (*p* ≤ 0.05).

**FIGURE 2:**
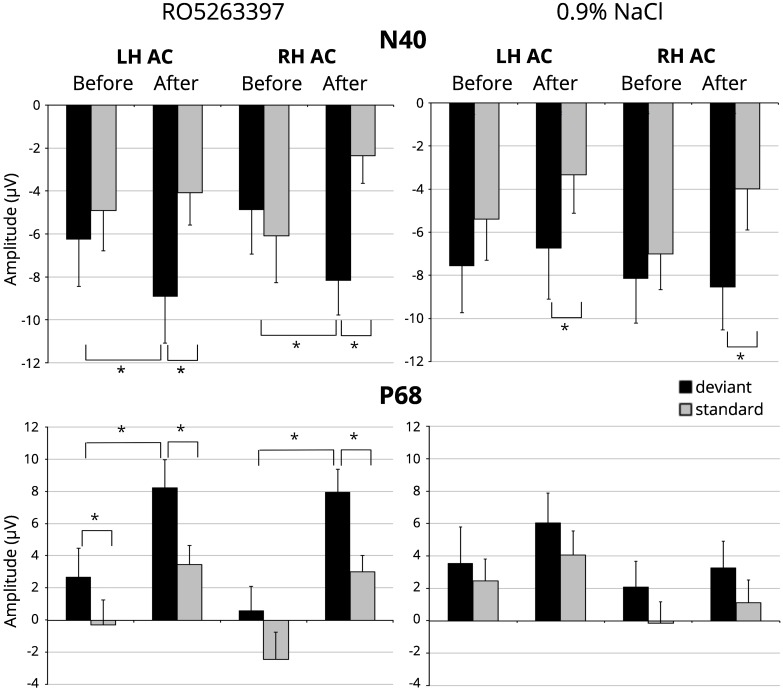
N40 and P68 amplitude in response to standard (gray columns) and deviant (black columns) stimuli before and after the TAAR1 agonist (RO 5263397) injection in left (LH AC) and right-hemisphere auditory cortex (RH AC). Error bars are standard error of the mean. Stars denote statistical significance (*p* ≤ 0.05).

Figure 1 illustrates the averaged difference responses obtained during the experiment. For the N40 component a significant *Stimulus type* main effect [*F*(1,21) = 7.059; *p* = 0.015] and *Injection* ×*Stimulus type* interaction [*F*(1,21) = 9.087; *p* = 0.007] in the RO5263397 administration condition was found. Fisher LSD *post hoc* test indicated significantly more negative response to the deviant stimuli after the TAAR1 agonist injection (*p* = 0.029) The difference between deviant and standard stimuli responses also became significant after the RO5263397 administration (*p* < 0.001) (Figure 2). MMN-like responses analysis revealed the significant *Injection* main effect [*F*(1,21) = 9.087; *p* = 0.007] indicating an increased difference between deviant and standard responses after the RO5263397 injection (Figure 1).

In saline condition a significant *Stimulus type* main effect [*F*(1,21) = 6.310; *p* = 0.020] was found. The paired sample *t*-test showed significantly larger mean amplitude to the deviant than to the standard stimulus after [left hemisphere: *t*(21) = -2.357, *p* = 0.028; right hemisphere: *t*(21) = -2.703, *p* = 0.013], but not before saline injection (Figure 2). It should be noted that there is a slight but not significant decrease [*F*(1,21) = 2.168; n.s.] in the response amplitude to the standard stimulus, which may reflect the stress effect caused by the fact of injection (Karl et al., 2006).

For the P68 component the significant main effects for *Stimulus type* [*F*(1,21) = 11.588; *p* = 0.003] and *Injection* [*F*(1,21) = 11.422; *p* = 0.003] were found. Paired *t*-tests confirmed larger amplitude in response to the deviant stimuli before [left hemisphere: *t*(21) = 2.103, *p* = 0.048] and after [left hemisphere: *t*(21) = 2.564, *p* = 0.018; right hemisphere: *t*(21) = 3.571, *p* = 0.002] the injection and an increased positivity in response to the standard [left hemisphere: *t*(21) = 2.376, *p* = 0.027; right hemisphere: *t*(21) = 3.772, *p* = 0.001], and deviant stimuli [left hemisphere: *t*(21) = 2.200, *p* = 0.039; right hemisphere: *t*(21) = 2.590, *p* = 0.017] after the RO5263397 administration (Figure 2). No difference in MMN-like responses before and after injection were found [*F*(1,21) < 1; n.s.]. No significant *Injection* or *Stimulus type* effects were revealed in saline condition.

No effects on the latency of any described components after injections after saline or RO5263397 were found.

## Discussion

The current study was aimed to evaluate the selective TAAR1 partial agonist RO5263397 effect on MMN-like responses in freely moving mice. Recorded ERPs contained two main components: negative – with 40 ms latency (N40) and positive – with 68 ms latency (P68). The pitch deviance-elicited changes in the control paradigm and before the injection were established for the P68 component. The MMN-like difference for the N40 component was also found after the TAAR1 agonist injection.

Currently, there is no consensus about the MMN-like response component in rodents that is considered to be analogous to human MMN. Auditory ERPs in humans consist of three main components that are defined by peak latencies and polarity: P50, N100, and P200. MMN arises in approximately 150–200 ms after the deviant stimulus in oddball paradigm and partly overlapped with the N100 component (Näätänen et al., 2011). The latency and polarity of rodent auditory ERPs are quite variable and strongly depend on the reference electrode position and anesthetic agents (Harms et al., 2016). Some authors consider that mouse auditory ERP latencies are approximately 40% of latencies seen in humans (Siegel et al., 2003) and that P1, N1, and P2 components in mice are analogous to the human P50, N100, and P200, according to the ISI response relationship (Umbricht et al., 2002, 2004; Maxwell et al., 2004). Many researchers report an MMN-like response in rodents (positive or negative), reaching a maximum between 50 and 100 ms (Ahmed et al., 2011; Astikainen et al., 2011; Nakamura et al., 2011; Jung et al., 2013; Shiramatsu et al., 2013; Harms et al., 2014; Sivarao et al., 2014; Ruusuvirta et al., 2015).

Event-related potentials registered in our study were highly similar in polarity and peak latencies to components obtained in rats (Jung et al., 2013; Aleksandrov et al., 2018) and mice (Umbricht et al., 2004; Ehrlichman et al., 2008). After the RO5263397 administration the N40 amplitude increased in response to deviant stimuli, which led to the MMN-like difference in the 36–44 ms time interval. Besides the P68 amplitude was increased in response both to standard and deviant stimuli and no difference for the MMN-like response was found.

Mismatch negativity is considered a correlate of pre-attentive processes and associated with a change detection due to a mismatch in echoic memory traces (Näätänen et al., 2011). The human MMN is a well-known schizophrenia biomarker (Turetsky et al., 2007; Light and Swerdlow, 2015). In patients with schizophrenia, the MMN reduced amplitude is a stable indicator of anomaly in hearing and attention (Javitt et al., 1993; Michie, 2001; Light and Braff, 2005). A gradual decrease in the MMN amplitude reflects cognitive and functional dysfunctions, depending on the disease duration (Näätänen and Kähkönen, 2009).

Impaired MMN generation is often associated with glutamatergic NMDA receptor system deficiency. Some research demonstrate that the competitive and non-competitive NMDA antagonists reduce MMN amplitude in humans (Javitt et al., 1996; Umbricht et al., 2000; Kreitschmann-Andermahr et al., 2001) and rats (Tikhonravov et al., 2008; Harms et al., 2018). Deviance-elicited change in N40 amplitude in mice is also decreased under the NMDA antagonist ketamine action that makes it similar to MMN in humans (Ehrlichman et al., 2008). Furthermore, other compounds with NMDA antagonist properties (alcohol and nitric oxide) also reduced MMN (Jääskeläinen et al., 1995; Pang and Fowler, 1999).

Recent studies provide evidence that the NMDA receptors composition and functions may depend on TAAR1. *Taar1* knockout mice demonstrate the reduced NMDA receptor subunits expression and phosphorylation in prefrontal cortex and striatum (Espinoza et al., 2015; Sukhanov et al., 2016). In addition, TAAR1 agonists are able to suppress the hyperlocomotion induced by the NMDA antagonist (Revel et al., 2011, 2013), decrease impulsive behavior in mice (Espinoza et al., 2015) and reverse the cognitive impairments in rats treated with the glutamate NMDA receptor antagonist (Revel et al., 2013). Thus, the TAAR1 activation is able to modulate directly or indirectly the NMDA-mediated glutamatergic activity, likely ameliorating the hypoglutamatergic state. That may be the possible explanation of increased deviance-elicited change in N40 amplitude after the TAAR1 agonist administration.

The monoaminergic systems, being the negative TAAR1 neuromodulation targets, may also modulate the NMDA activity, thus playing a potential role in MMN generation (Espinoza et al., 2011; Revel et al., 2011). Otherwise, numerous studies have shown the multidirectional effect of dophaminergic and serotonergic systems on MMN. As reported by Kähkönen et al. (2001) a decrease in the dopaminergic activity after the D2 receptor antagonist (haloperidol) administration in humans leads to an increase in the MMN amplitude. However, some other studies have found no effects of haloperidol (Kähkönen et al., 2002; Pekkonen et al., 2002) or selective D2 agonist (bromocriptine) and D1/D2 agonist (pergolide) (Leung et al., 2007) on MMN. In a number of studies, a 5-HT synthesis decrease by the acute tryptophan depletion (ATD) has been found to impair the MMN amplitude (Ahveninen et al., 2002). A negative component decrease in the oddball paradigm has also been found in the 50% serotonin depletion condition after the para-chlorophenylalanine administration in rats (Ehlers et al., 1991). On the contrary, some studies in humans indicate an improved attention and executive functions (Coull et al., 1995; Schmitt et al., 2000; Murphy et al., 2002), an increased MMN amplitude and a decreased MMNm latency (Kähkönen et al., 2005) after the ATD. Although some studies show increased MMN amplitude with monoaminergic systems activity decrease, but the role of this system in the MMN generation remains unclear. Therefore, it is difficult to conclude that an increase in the MMN-like response after the TAAR1 agonist administration may be associated with dopaminergic or serotonergic activity neuromodulation.

The ERPs modulation can also be associated with the increased intensity dependence due to the reduced serotonergic neurotransmission (Hegerl and Juckel, 1993; Hegerl et al., 2001). Thus, an increase in the P68 amplitude in response to standard and deviant stimuli after the TAAR1 agonist administration may be the result of new afferents activation associated with the modulation of monoaminergic system.

Therefore, the TAAR1 activation, most likely acting thorough the NMDA receptor modulation, plays the critical role in the N40 MMN-like response increase. Taken together these data indicate that TAAR1 activation can significantly increase the MMN response suggesting the potential antipsychotic and pro-cognitive activity of TAAR1 agonists.

## Data Availability

The datasets generated for this study are available on request to the corresponding author.

## Ethics Statement

All animal studies were carried out in strict accordance with the guidelines of the Ministry of Health of Russian Federation and the principles of the Basel Declaration. All experiments were approved by the Saint Petersburg State University Ethical Committee for Animal Research (approval number 131-03-4 from March 14, 2016). All surgical procedures were performed under anesthesia, and all efforts were made to minimize suffering.

## Author Contributions

AA and RG provided the concept of the study. AA, RG, VK, and AV provided design of the study. VK, ED, AV, and NP contributed to the data acquisition and analysis. All authors contributed to the interpretation and discussion of the results, drafted, and critically revised the manuscript.

## Conflict of Interest Statement

The authors declare that the research was conducted in the absence of any commercial or financial relationships that could be construed as a potential conflict of interest.
